# The Role of Toxicology Investigations in Overdose Deaths

**DOI:** 10.7759/cureus.79352

**Published:** 2025-02-20

**Authors:** Matteo A Sacco, Saverio Gualtieri, Chara Spiliopoulou, Alessandro P Tarallo, Maria C Verrina, Isabella Aquila

**Affiliations:** 1 Department of Medical and Surgical Sciences, Magna Graecia University, Catanzaro, ITA; 2 Department of Forensic Medicine and Toxicology, National and Kapodistrian University of Athens School of Medicine, Athens, GRC; 3 Department of Medical and Surgical Sciences, Institute of Legal Medicine, Magna Graecia University, Catanzaro, ITA

**Keywords:** forensic toxicology, gc-ms, lc-ms/ms, overdose, synthetic opioids, toxic substances

## Abstract

Overdose involves the administration of one or more narcotic substances in quantities exceeding the body's tolerance threshold, leading to toxic effects ranging from mild to fatal. The clinical manifestations of an overdose vary depending on the toxic substance's specific molecular action, such as stimulation or suppression of the nervous system. Common toxic agents include synthetic opioids like fentanyl, cocaine, barbiturates, benzodiazepines, and cannabinoids. This study emphasizes the critical role of forensic toxicology in identifying overdose deaths, focusing on the molecular mechanisms of toxicity, post-mortem redistribution, and the interpretation of toxicological findings. Advanced methodologies such as gas chromatography-mass spectrometry (GC-MS) and liquid chromatography-tandem mass spectrometry (LC-MS/MS) are discussed as pivotal tools for identifying toxic substances and their metabolites. Biological matrices such as blood, urine, vitreous humor, and organ tissues are evaluated for their utility in toxicological investigations. Accurate interpretation of results informs not only the cause of death but also patterns of substance abuse, contributing to the development of preventive strategies. This study highlights the growing complexity of psychoactive substances, emphasizing the necessity for precise and innovative toxicological techniques in forensic practice.

## Introduction and background

Drug overdose

Drug overdose is a major global health concern, contributing to a substantial number of fatalities annually. The increasing prevalence of synthetic opioids, such as fentanyl and its analogs, has exacerbated the crisis, leading to a surge in drug-related deaths worldwide. According to the United Nations Office on Drugs and Crime (UNODC), opioid overdose deaths have reached unprecedented levels, particularly in North America and parts of Europe. The challenge of addressing overdose-related fatalities is further complicated by the emergence of novel psychoactive substances (NPS), which often evade routine toxicological screening.

Forensic toxicology plays a crucial role in investigating drug-related deaths by identifying and quantifying toxic substances in biological specimens. Advances in analytical techniques, including mass spectrometry-based methods, have significantly improved the detection of drugs in postmortem cases. However, several challenges remain, particularly in the interpretation of toxicological findings and the differentiation between fatal intoxications and incidental drug presence. Previous studies have emphasized the importance of comprehensive toxicological screening in forensic investigations, yet gaps persist in the identification of newly emerging substances and the standardization of postmortem toxicological protocols.

Toxicology investigations serve a critical function in the realm of forensic science, providing essential insights into the presence and impact of substances within biological samples. Forensic toxicology is defined as the analysis of biological samples to detect toxins, including drugs, thereby assisting in the judicial process. This analysis not only aids in determining whether substances contributed to a person's death but also supports legal proceedings by establishing evidence of substance involvement. These investigations are pivotal in cases of overdose deaths as they can reveal the type and concentration of substances present, offering a comprehensive understanding of their potential role in the fatal event. By identifying toxic substances, toxicology investigations help piece together the circumstances surrounding an overdose, informing both medical and legal conclusions.

Drug overdoses frequently involve a variety of substances, many of which are commonly detected in toxicology investigations [1-3]. Toxicology reports can highlight the specific drugs involved, providing insight into trends and patterns in drug use that may inform public health responses and preventative strategies. Understanding the types of substances commonly involved in overdoses is crucial for developing effective interventions and policies aimed at reducing the incidence of such fatalities. Postmortem toxicology plays a crucial role by suggesting drug intoxication, highlighting the need for further studies, and guiding toxicological analyses [4]. By integrating toxicological findings with data from the overdose scene and witness accounts, investigators can more accurately identify the specific drugs contributing to an overdose death [5]. This comprehensive approach not only enhances the understanding of the circumstances leading to the death but also assists in identifying potential legal and public health actions. Toxicology investigations thus provide a vital link between forensic science and the legal system, helping to ensure that justice is served and that preventive measures are informed by accurate and detailed data.

Overdose is the administration of one or more narcotic substances in excessive doses that exceed the body's tolerance threshold, causing more or less serious toxic side effects [6]. Overdose can manifest in various ways depending on the type of drug that excites or depresses the nervous system. Furthermore, it causes serious impairment of vital functions and can lead to death [7]. Substances that can cause an overdose include both illicit drugs and prescribed medications. Among the most common substances are cocaine, heroin, fentanyl, ecstasy, barbiturates, benzodiazepines, methamphetamines, and cannabinoids. This study underscores the importance of forensic investigations in identifying overdose deaths. Overdose deaths have increased as a result of the widespread availability of powerful synthetic opioids, such as fentanyl, which is now globally prevalent. According to the United Nations Office on Drugs and Crime (UNODC), synthetic opioids, primarily fentanyl and its analogs, have been responsible for a significant rise in drug-related fatalities worldwide. In the United States alone, the Centers for Disease Control and Prevention (CDC) reported that synthetic opioid-related deaths accounted for over 70,000 fatalities in 2021. Similarly, Canada and parts of Europe have witnessed alarming increases, with fentanyl overdoses representing a substantial portion of drug-related deaths. The global nature of this crisis underscores the urgent need for coordinated public health interventions and regulatory measures [8]. The study and identification of these opioids in various postmortem samples and the correct interpretation of toxicological data are essential to developing and implementing preventive strategies [9]. The samples used for toxicological analysis include blood, urine, vitreous humor, liver, and brain. Several factors must be analyzed, such as chemical properties, essential pharmacokinetic parameters, and postmortem body redistribution [6]. A study examined routine drug screening data in 2996 forensic autopsy cases over 18.5 years [9]. Drug screening was performed using gas chromatography/mass spectrometry (GC/MS) in all cases. Drugs were detected in 486 cases (16.2%), including amphetamines, major and minor tranquilizers (n=294), antidepressants (n=21), cold remedies, and more. Among these cases, fatal intoxication (n=123) mostly involved amphetamines (n=73), major tranquilizers (n=37), and minor tranquilizers (n=86) [2]. Overdose was caused, in almost all cases, by self-administration of the substances, but the presence of these substances was also detected in cases of homicide [10].

These evaluations highlight the effectiveness of systematic routine toxicological analysis in investigating not only the cause of death but also the background of deceased individuals. Extensive toxicological screening is therefore necessary for managing social and forensic risks, given the wide variety of existing drugs.

Collection and analysis of biological samples

Various types of biological samples are utilized, each offering unique insights into the individual's exposure to substances. Commonly used samples for toxicological analysis include blood, urine, and tissues such as the liver, brain, and kidneys. Additionally, stomach and gastric contents are frequently analyzed, as they can provide crucial information on recent substance ingestion and potential toxic exposures. Blood samples, particularly from the femoral vein, are highly valued due to their reliability in reflecting the concentration of substances at the time of death [11]. Urine samples, on the other hand, are useful for detecting drugs or their metabolites that may have been present over a longer period before death [4]. Tissues like the liver can provide information on the body's metabolic response to drugs, offering additional context to the toxicological findings [4]. The choice of sample depends on the availability and condition of the body, as well as the specific substances suspected to be involved in the death.

The collection of biological samples during an autopsy is a meticulous process that requires adherence to established protocols to ensure the integrity of the samples. Autopsy procedures involve both traditional and advanced methods to obtain necessary specimens. Traditional methods include direct sampling from organs and bodily fluids, while imaging techniques are increasingly used to guide and supplement sample collection [11]. The autopsy must be comprehensive to avoid missing vital evidence; this includes obtaining samples from various organs and tissues to provide a complete toxicological profile. Careful documentation and chain-of-custody practices are essential throughout the process to preserve the legal and scientific value of the samples [12]. The collected samples serve as the foundation for subsequent toxicological analysis, making their accurate collection a critical step in the investigation of overdose deaths.

Analyzing toxicological evidence requires sophisticated techniques and equipment to detect and quantify substances present in biological samples. Forensic toxicologists employ a range of analytical methods, including gas chromatography-mass spectrometry (GC-MS) and liquid chromatography-mass spectrometry (LC-MS), which are considered gold standards in the field [13]. These techniques allow for the precise identification and measurement of drugs and their metabolites, providing crucial data for determining the cause of death. The role of forensic toxicologists extends beyond mere analysis; they must also interpret the results in the context of the case, considering factors such as the individual's medical history and potential drug interactions. This comprehensive approach ensures that toxicological findings contribute effectively to understanding the circumstances surrounding an overdose death.

This study aims to address these gaps by providing an in-depth analysis of overdose-related deaths, focusing on the detection and interpretation of toxic substances. By examining a range of biological specimens and utilizing advanced analytical methods, this research contributes to the ongoing efforts to improve forensic toxicology practices and inform public health interventions.

## Review

Materials and methods

This systematic review was conducted following the PRISMA (Preferred Reporting Items for Systematic Reviews and Meta-Analyses) guidelines using the PubMed search engine.

**Figure 1:**
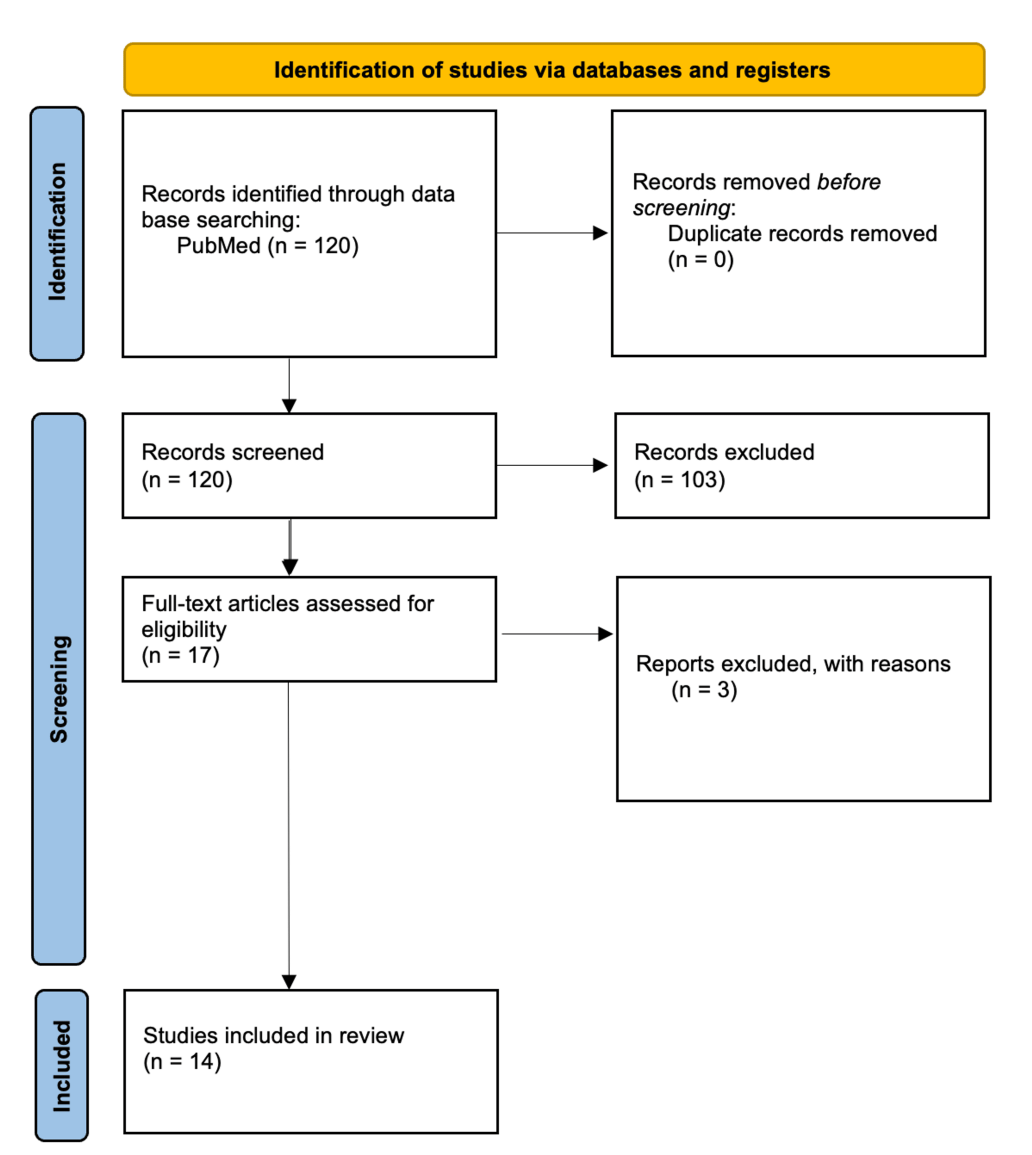
PRISMA flow diagram PRISMA - Preferred Reporting Items for Systematic reviews and Meta-Analyses

The keywords used were "drug overdose" AND "forensic" AND "toxicological investigations". The data were extracted from peer-reviewed articles, case reports, and retrospective analyses related to drug-related deaths. A total of 120 results were evaluated, excluding works focused on toxicological investigations in living subjects or those lacking forensic relevance. Additionally, the references cited in the included articles were cross-referenced to identify other pertinent studies. The article selection process was organized into four main phases: identification, screening, eligibility, and inclusion. Titles and abstracts identified during the search phase were evaluated for inclusion. Duplicate articles, non-relevant studies, and those not in English were excluded. Selected articles were thoroughly examined to assess their relevance and methodological quality. Eligibility criteria included: 1) studies analyzing drug-related deaths with detailed toxicological data, 2) use of advanced analytical methods such as LC-MS/MS and GC-MS, 3) quantitative data on blood concentrations and postmortem distributions, and 4) analyses of pharmacological interactions. A total of 14 studies meeting the above criteria were included in Table 1. Data extracted from the articles included study objectives, methods, main results, and conclusions.

**Table 1:** Analysis of studies from literature review Table credits: authors Matteo A. Sacco and Isabella Aquila LC-MS/MS - liquid chromatography-tandem mass spectrometry; NPS - novel psychoactive substances; LC-QTOF-MS - quadrupole time-of-flight liquid chromatography-tandem mass spectrometry; GC-MS - gas chromatography-mass spectrometry; PMA - paramethoxyamphetamine; MDMA - 3,4-methylenedioxymethamphetamine; HILIC-MS - hydrophilic interaction chromatography-mass spectrometry; CSF - cerebrospinal fluid

Study title	Authors	Study objective	Main methods	Main results	Conclusions
Fentanyl-related deaths in Ontario, Canada: toxicological findings and circumstances of death in 4395 cases (2020–22)	Adamo et al. [14]	Investigate fentanyl-related deaths in Ontario from 2020 to 2022 to analyze toxicological trends, demographic factors, causes and manners of death, as well as the concomitant use of other substances.	Retrospective study with toxicological analysis of 4395 cases using LC-MS/MS to quantify fentanyl (>1.3 ng/ml) from femoral blood or clinical samples	Fentanyl was present in 69% of mixed toxicity cases and 19% of fentanyl intoxications. Cocaine and methamphetamine were common in mixed cases.	The analyses provide crucial data on fentanyl trends and suggest increased stimulant use in combination with opioids. The combined use of fentanyl with stimulants and alcohol increases the risk of overdose.
Lethal vortioxetine poisoning? A forensic investigation	Zuccarello et al. [15]	Investigate a case of possible lethal vortioxetine poisoning through toxicological and histological analyses.	Toxicological analysis of biological samples from femoral blood, brain, liver, kidney, and lungs using LC-MS/MS and histopathological investigation of a specific case.	Vortioxetine was present at levels 35-135 times higher than therapeutic values in femoral blood. Microscopic changes were indicative of fatal terminal arrhythmia and chronic ischemic cardiomyopathy.	Lethal vortioxetine levels suggest a mechanism of cardiotoxicity not yet well-documented in existing literature.
Strategic Decision-Making by a Forensic Toxicology Laboratory in Response to an Emerging NPS: Detection, Quantitation and Interpretation of Carfentanil in Death Investigations in Ontario, Canada, July 2017 to June 2018	Solbeck et al. [16]	Describe strategies for detecting and quantifying carfentanil and interpreting its presence in death investigations.	Modification of existing LC-MS/MS, LC-QTOF-MS, and GC-MS methods to include carfentanil and analysis of over 4953 cases.	Carfentanyl is identified in 160 postmortem cases, with blood concentrations ranging from <0.1 to 9.2 ng/mL.	Incorporating carfentanil into standard methods improved surveillance and interpretation in medicolegal investigations.
Postmortem Brain–Blood Ratios of Codeine, Fentanyl, Oxycodone and Tramadol	Nedahl et al. [17]	Examine brain-to-blood ratios for codeine, fentanyl, oxycodone, and tramadol in postmortem cases.	Quantification of drugs in blood and brain tissue using solid-phase extraction and LC-MS/MS from 210 autoptic cases.	Brain-to-blood ratios ranged from 0.29 to 16 for fentanyl and from 0.47 to 4.6 for codeine, from 0.11 to 6.0 for oxycodone, from 0.047 to 6.8 for tramadol, demonstrating specific distributions.	The data support using brain tissue as an alternative to blood for toxicological analyses in complex cases.
Brain-blood ratio of morphine in heroin and morphine autopsy cases	Nedahl et al. [18]	Investigate morphine concentrations and brain-to-blood ratios in heroin and morphine autopsy cases.	Analysis of 98 autopsy cases with morphine quantification using LC-MS/MS from femoral blood and brain tissue.	Average brain-to-blood ratios were 1.2 for morphine and 1.8 for heroin cases; ratios varied based on cause of death. 6-AM and noscapine can help identify heroin use in cases where the brain-to-blood ratio is inconclusive.	Brain tissue analysis can supplement blood toxicology, but brain-to-blood ratios alone cannot distinguish heroin from morphine use.
The effects of the (fentanyl-fueled) drug overdose epidemic on medicolegal death investigation in the United States	Davis et al. [19]	Examine the impact of the fentanyl-fueled overdose epidemic on medicolegal death investigations in the United States.	Analysis of public data and medicolegal reports to examine trends in deaths and investigative resources.	The fentanyl epidemic increased overdose deaths by 31% in 2020 compared to 2019 and by 14% in 2021 compared to 2020.	The increase in overdose deaths requires investments in medicolegal resources to ensure timely and accurate investigations.
Postmortem Toxicology of New Synthetic Opioids	Concheiro et al. [20]	Review postmortem toxicology of synthetic opioids, including fentanyl and derivatives.	Literature review peer-reviewed postmortem data on synthetic opioids using LC-MS/MS focusing on pharmacokinetics and redistribution, concentrations of post-mortem blood, brain, liver, tumor, and urine samples.	Highlighted the complexities of interpreting synthetic opioid concentrations due to redistribution and pharmacokinetics	Potency and postmortem redistribution must be considered to accurately interpret postmortem levels.
Commentary: Fentanyl-related death and the underreporting risk	D'Errico [21]	Examine the challenges in toxicological analysis and underreporting of fentanyl-related deaths.	Literature review of toxicological and forensic analysis of fentanyl postmortem data and its redistributions, emphasizing the role of norfentanyl.	Postmortem fentanyl concentrations vary significantly due to redistribution; norfentanyl ratios help assess acute toxicity. A high fentanyl/norfentanyl ratio (>8) suggests acute intoxication, while a ratio <2.5 indicates chronic use.	Better toxicology infrastructure is needed to address fentanyl underreporting and improve forensic investigations.
Medicolegal aspects of PMA-related deaths	Rojek et al. [22]	Analyze three fatal PMA poisoning cases and their medicolegal implications.	Case reports of fatal PMA poisonings including autopsy findings, toxicology using LC-MS/MS, and contextual analysis.	High PMA concentrations (10–27 mg/L) in all three cases; acute cardiorespiratory failure, pulmonary edema, and multi-organ failure were identified as the cause of death.	The low threshold between toxic and lethal doses makes PMA extremely dangerous, especially when combined with other drugs like MDMA or amphetamine.
Hair testing in postmortem diagnosis of substance abuse: An unusual case of slow-release oral morphine abuse in an adolescent	Baillif-Couniou et al. [23]	Investigate slow-release oral morphine abuse in a teenager using hair analysis and other postmortem methods.	Segmental hair analysis combined with blood, urine, gastric content and umor and tissue toxicology using chromatographic techniques and LC-MS/MS.	Hair analysis confirmed regular morphine exposure over the years (from 131 to 250 pg/mg); total morphine in blood reached toxic levels (584 ng/mL).	The value of hair analysis provides a broader time window compared to blood and urine, documenting chronic use and helping to clarify the circumstances of death.
An update on oxycodone: lessons for death investigators in Australia	Pilgrim et al. [24]	Explore oxycodone-related deaths in Australia, focusing on prescribing practices and coronial investigations.	Analysis of 806 oxycodone-related deaths using the National Coronial Information System, including toxicology and prescribing data.	Oxycodone-related deaths increased sevenfold from 2001 to 2011, only oxycodone (11.8%) or mixed drugs (63.4%), with mental illness and chronic pain common in cases. 24% of prescriptions were inappropriate, often for migraine.	The increase in oxycodone prescriptions is directly correlated with the rise in deaths related to this substance. Physicians must exercise greater caution in patient selection and dosing.
Drug poisoning deaths in Sweden show a predominance of ethanol in mono-intoxications, adverse drug-alcohol interactions, and poly-drug use	Jones et al. [25]	Analyze drug poisoning deaths in Sweden over 10 years, focusing on ethanol mono-intoxications and poly-drug interactions.	Retrospective analysis of 6894 forensic autopsies in Sweden from database "TOX BASE" of the National Board of Forensic Medicine with toxicology results from femoral blood and urine samples using LC-MS and GC-MS.	Most drug poisoning deaths involved poly-drug use; ethanol was the leading substance in mono-intoxications (63%).	Comprehensive toxicology and multi-source data are essential for determining the cause and manner of drug poisoning deaths.
A Fatal Overdose of Cocaine Associated with Coingestion of Marijuana, Buprenorphine, and Fluoxetine	Giroud et al. [26]	Investigate a fatal cocaine overdose involving marijuana, buprenorphine, and fluoxetine.	Toxicological analysis using HILIC-MS to quantify cocaine and its metabolites in biological fluids (blood, urine, CSF, bile) and tissues (brain, muscles, and liver).	High cocaine concentrations detected in blood (5.0 mg/L) and brain(21.2 mg/Kg); cannabis and fluoxetine may have exacerbated toxic effects.	Interactions between cocaine and co-ingested substances can exacerbate toxic effects, underscoring the need for thorough toxicology.
A critical review of the causes of death among post-mortem toxicological investigations: analysis of 34 buprenorphine-associated and 35 methadone-associated deaths	Pirnay et al. [27]	Examine the role of buprenorphine and methadone in 69 toxicological deaths in Paris.	Retrospective analysis of postmortem toxicology, autopsy findings from blood and urine using GC-MS, and police reports for buprenorphine and methadone cases.	Buprenorphine was implicated in 12% of cases and methadone in 9%; toxic or lethal concentrations were common.	Understanding the role of substitution drugs in fatalities is critical for improving treatment safety and forensic investigations. Other substances, such as alcohol and benzodiazepines, play a significant role in the lethal process.

Results of the literature review

From the literature review, it emerged that the use of substances of abuse represents a growing social problem in several countries worldwide. This issue is particularly concerning given the increase in molecular varieties of substances, which are becoming progressively more dangerous, and the rising frequency of abuse and lethality [28].

In overdose deaths, scene inspection is a cornerstone for determining the state of the premises, identifying substances and materials used for their administration, and significantly aiding forensic investigations. In such circumstances, substances or their traces are often found near the body [29]. Additionally, it is common to find different types of substances at the same inspection site, confirming the prevalence of poly-substance abuse. Cataloging and recording the various molecules detected is crucial to discriminating those actually involved in the death. One of the main powerful synthetic opioids is fentanyl, which is now globally prevalent. Some studies emphasize this argument: Adamo et al. reported that fentanyl was present in 69% of mixed toxicity cases in Ontario, with cocaine and methamphetamine being the most commonly associated drugs [14]; D'Errico described the different concentrations of fentanyl/norfentanyl ratio to suggest acute toxicity or chronic use [21]. Year by year, the fentanyl correlated deaths increased: Davis et al. showed increased overdose deaths by 31% in 2020 compared to 2019 and by 14% in 2021 compared to 2020 [19]. Similarly, any traces of blood, syringes, tourniquets, teaspoons, and other tools used for substance administration must be carefully cataloged, stored, and analyzed. Finally, the presence of interventions or third-party involvement at the scene must be evaluated and excluded, as such evidence raises questions about potential procured overdose.

Autopsy remains the "gold standard" for evaluating overdose deaths. While scene findings may highlight the presence of substances of abuse, autopsies evaluate their actual impact on the cause of death and identify potential additional causes. Findings from this study demonstrate how alcohol frequently represents one of the primary substances involved in violent deaths and is often detected in combination with other substances in poly-intoxications [30].

In industrialized countries, opiates are the main etiological agents in overdose deaths, with a significant increase in their frequency. Solbeck et al. identified carfentanil in 160 postmortem cases, with concentrations below 0.1 ng/mL [16]. Pilgrim et al. highlighted a sevenfold increase in oxycodone-related deaths between 2001 and 2011 [25]. The primary mechanism involved is acute respiratory failure, with autopsy findings often showing liquid in the respiratory system, cerebral edema, and pulmonary edema [25]. These substances of abuse act on the endogenous opioid system, exerting psychotropic effects through the mu receptors. Activation of these receptors triggers a complex intracellular cascade, promoting dopamine release, inhibiting pain mechanisms, and inducing euphoria. In overdose cases, excessive stimulation of the pontine brain region, which regulates breathing, leads to the aforementioned respiratory depression [25].

The primary receptors involved in the action of opiates are: 1) Mu receptors, including mediate analgesia, euphoria, sedation, respiratory depression, reduced gastrointestinal motility, and physical dependence. They also alter medullary responses to hypercapnia and decrease respiratory drive to hypoxia, resulting in ineffective respiratory responses [31]. 2) Kappa receptors, including mediate analgesia, diuresis, miosis, and dysphoria [31]. And 3) Delta receptors, including mediate analgesia, inhibition of dopamine release, and suppression of the cough reflex [31].

It is also important to consider emerging drugs of abuse, including synthetic cannabinoids, synthetic cathinones, novel psychoactive substances (NPS), and anabolic steroids. Synthetic cannabinoids, often marketed as 'Spice' or 'K2', mimic the effects of THC but pose significant health risks, including psychosis and cardiovascular complications. Synthetic cathinones, commonly known as 'bath salts', have been associated with severe agitation, paranoia, and cardiovascular toxicity. Additionally, novel psychoactive substances continue to evolve rapidly, complicating detection and regulation. Anabolic steroids, although primarily used to enhance performance, are frequently misused and can lead to severe psychiatric and cardiovascular effects. The increasing prevalence of these substances underscores the need for continuous monitoring and updated toxicological assessments.

Stimulants such as cocaine and chemically related substances inhibit the reabsorption of noradrenaline and dopamine, causing significant increases in bioavailability. Giroud et al. explored toxic interactions involving cocaine, fluoxetine, and cannabinoids [26]. This leads to a sudden increase in systemic blood pressure and the onset of potentially fatal tachyarrhythmias, which are confirmed at autopsy by findings of myocardial infarction or stroke. Other substances lead to cardiotoxicity: Zuccarello et al. reported the cardiotoxicity of vortioxetine at levels 35-135 times therapeutic thresholds [15]. The presence of risk factors typical of substance abusers, such as smoking and alcohol consumption, as well as endogenous factors like atherosclerotic disease or uncontrolled hypertension, further increase the risk.

Benzodiazepines, another common class of substances involved in overdose deaths, enhance the inhibitory effect of gamma-aminobutyric acid (GABA), producing sedative, hypnotic, anxiolytic, anesthetic, anticonvulsant, and muscle relaxant effects [32]. When combined with ethanol, they primarily cause respiratory failure, which can be fatal if not promptly treated. In Pirnay et al. study, the association of benzodiazepine, alcohol, buprenorphine, and methadone plays a significant role in the lethal process [27]. In cases of suspected overdose, toxicological tests are necessary to confirm the hypothesis and determine whether an exogenous substance caused or contributed to the death [8, 32]. Jones et al. (2010) emphasized the predominant role of ethanol in mono-intoxication cases, with mean concentrations of 3.06 g/L [25].

The main biological matrices in forensic toxicology are typically body fluids such as bile, gastric contents, blood, vitreous humor, and urine. A particular sample is the hair; Baillif-Couniou et al. demonstrated how segmental hair analysis could provide a broader temporal perspective, documenting chronic use of substances such as slow-release morphine [23].

However, it is also recommended to collect fragments of organs such as the liver, lungs, brain, and kidneys, as exogenous substances can often be detected in these tissues. In two articles, Nedahl et al. demonstrated the utility of brain-to-blood ratios for interpreting postmortem opioid distributions, with ratios ranging from 0.29 to 16 for fentanyl, from 0.47 to 4.6 for codeine, from 0.11 to 6.0 for oxycodone, from 0.047 to 6.8 for tramadol, demonstrating specific distributions [17-18]. 

In cases where biological matrices are unavailable due to factors such as decomposition, samples from muscle, bone marrow, or bone fragments can be analyzed as alternatives. Studies have shown significant differences in drug concentrations sampled from various anatomical points, so it is advisable to collect as much material as possible during autopsy to ensure the reproducibility of investigations [28-30].

Techniques used in forensic toxicology

Techniques used in toxicological investigations are categorized into first- and second-level analyses. First-level analyses are screening tests, typically employing immunoassay techniques due to their low cost and ability to evaluate multiple substances simultaneously [27]. If a substance is detected above the established cut-off level, confirmatory tests are required. Confirmatory analyses use advanced techniques such as mass spectrometry, often combined with gas chromatography (GC-MS) or liquid chromatography (LC-MS), which target single substances with high specificity [27].

Gas chromatography-mass spectrometry (GC-MS) and liquid chromatography-tandem mass spectrometry (LC-MS/MS) are two fundamental techniques in forensic toxicology, valued for their high sensitivity and specificity. GC-MS is particularly effective in identifying volatile and semi-volatile compounds such as alcohols, amphetamines, and benzodiazepines [6-17]. The availability of extensive spectral libraries makes it an indispensable tool for the rapid identification of unknown compounds, while its ability to separate and quantify complex mixtures highlights its analytical efficiency. However, GC-MS has certain limitations, including its ineffectiveness in analyzing non-volatile, polar, or thermolabile compounds, which may degrade at the high temperatures required for gas chromatography [6-17]. Additionally, certain analytes require a derivatization step to enhance detection, increasing sample preparation time.

On the other hand, LC-MS/MS provides exceptional versatility in the analysis of non-volatile, polar, and thermolabile substances, such as synthetic opioids and their metabolites [31-32]. The tandem mass spectrometry component offers outstanding sensitivity, allowing the detection of trace levels of compounds in complex biological matrices. Moreover, LC-MS/MS does not require derivatization, which significantly reduces sample preparation time and enhances its applicability in high-throughput settings. Despite its advantages, LC-MS/MS presents challenges, such as higher operational costs and the relative scarcity of extensive spectral libraries for certain compounds, necessitating the development of custom methods [29-34].

In forensic toxicology, the comparative effectiveness of these methods often depends on the context. GC-MS is frequently preferred for the detection of volatile substances or routine drug screening in matrices such as urine. LC-MS/MS, however, is invaluable for the analysis of novel psychoactive substances, synthetic opioids, and metabolites that might be overlooked by GC-MS [29-33]. These two techniques are often employed in a complementary manner, with LC-MS/MS gaining prominence due to its ability to address the challenges posed by emerging drug classes and its rapid, comprehensive analytical capabilities. Together, they provide a robust framework for identifying and quantifying a wide array of substances, ensuring a comprehensive approach to forensic investigations.

To ensure legally valid results, strict quality standards are necessary. These include rigorous chain-of-custody procedures, personnel training, validation of analytical methodologies, and operational protocols for managing pre- and post-analytical phases. Additionally, proper calibration and maintenance of analytical instruments are critical for achieving accurate results.

In living subjects, urine is often the preferred biological matrix for detecting substances of abuse. This choice offers advantages such as non-invasive collection, large sample volumes, and the ability to detect substances or their metabolites days after ingestion. However, urine has limitations in clinical relevance for qualitative analysis due to variations in analyte concentration influenced by factors like dose, route of administration, time elapsed between intake and collection, and the individual's physical condition.

While GC-MS and LC-MS remain cornerstone techniques in toxicological analysis, additional advanced methodologies have gained prominence. Ultra-performance liquid chromatography-mass spectrometry (UPLC-MS) offers higher resolution and sensitivity compared to conventional LC-MS, allowing for faster and more precise analyses. Surface-enhanced Raman spectroscopy (SERS) provides a powerful tool for trace-level detection of toxic substances with minimal sample preparation. Nuclear magnetic resonance (NMR) spectroscopy is increasingly utilized for structural elucidation of unknown compounds, while isotope ratio mass spectrometry (IRMS) aids in the identification of endogenous versus exogenous substances in doping and forensic cases.

Furthermore, techniques like direct analysis in real time (DART) provide rapid, on-site detection of poisons and drugs, eliminating the need for extensive sample preparation. This is particularly valuable in forensic and emergency settings where immediate results are critical for medical or legal decision-making. The integration of these advanced techniques enhances the accuracy and efficiency of toxicological investigations.

Interpretation of toxicology results

Understanding toxicology report findings is critical for determining the role of substances in overdose deaths. These reports provide detailed information about the presence and concentration of drugs and poisons in biological samples. Postmortem toxicology results are instrumental in establishing both the cause and manner of death, especially in cases of suspected drug overdoses [13]. Toxicology involves not just detecting substances but understanding their potential impact on an individual's health and performance at the time of death [28]. Comprehensive analyses elucidate the circumstances surrounding an individual's demise, thereby supporting legal and medical examinations.

Interpreting drug concentrations in toxicology reports presents challenges due to factors such as body decomposition and postmortem redistribution of drugs [29, 30]. These factors can lead to variations in drug concentrations, complicating the determination of levels present at the time of death. Individual tolerance levels and the presence of multiple substances further complicate interpretation. Toxicologists must critically consider these influences to ensure accurate and reliable results.

Quantitative methods are essential in forensic toxicology for determining the concentration of substances in biological matrices and interpreting their potential effects. The interpretation of these concentrations requires an understanding of established thresholds that differentiate therapeutic, toxic, and lethal levels, which vary significantly depending on the substance, the matrix analyzed, and individual factors such as tolerance and metabolism [14-27].

For many substances, therapeutic ranges are well-documented and serve as a baseline for clinical use. For instance, therapeutic levels of morphine in blood typically range between 0.02 and 0.1 mg/L [16-17]. Concentrations exceeding this range, particularly above 0.2 mg/L, are considered toxic and may indicate misuse or overdose. Similarly, therapeutic levels of benzodiazepines like diazepam are generally below 0.5 mg/L, while toxic effects may appear at concentrations above 2 mg/L, with lethal outcomes often exceeding 5 mg/L in combination with other depressants such as alcohol [16-17].

However, interpreting these thresholds becomes challenging in postmortem toxicology due to factors such as postmortem redistribution, where concentrations in central blood samples may not accurately reflect ante-mortem levels. For example, lipophilic drugs like fentanyl may exhibit significantly higher concentrations in postmortem central blood due to redistribution from tissues. In these cases, peripheral blood samples, when available, provide a more reliable representation of ante-mortem drug levels [23-25].

Novel psychoactive substances (NPS) and synthetic opioids present additional complexities, as their thresholds are not as well-established [13]. For example, lethal concentrations of fentanyl in blood are often cited as above 3 ng/mL, but cases involving lower levels have been reported due to synergistic effects with other substances. Similarly, the toxicological profile of NPS, such as synthetic cannabinoids or designer stimulants, can vary widely, requiring careful interpretation of concentrations relative to the case context and other toxicological findings [13].

To ensure accurate differentiation between therapeutic, toxic, and lethal concentrations, it is crucial to consider not only the measured levels but also other case-specific factors, including the presence of polydrug use, the individual's medical history, and the timeline of substance administration. Additionally, the use of complementary matrices, such as liver or vitreous humor, can provide valuable insights into drug distribution and metabolism, aiding in the interpretation of concentration ranges.

Identifying drug interactions and overdose patterns

In the realm of postmortem toxicology, understanding commonly encountered drug interactions is crucial for accurately determining the causes of overdose deaths. Synergistic effects, where combined drugs produce a greater effect than their individual impacts, are frequently observed in toxicology investigations, particularly with substances like fentanyl and tramadol [35-37]. These interactions can complicate the toxicological analysis as they may alter the expected outcomes of individual drug effects. Toxicologists must be diligent in identifying these interactions to provide accurate interpretations of toxicological data. This vigilance ensures that underlying interactions do not obscure the true cause of death, highlighting the critical role of toxicology in forensic investigations.

Identifying patterns in substance use through toxicology is an essential aspect of overdose death investigations. Toxicologists employ a range of techniques to detect and analyze substances, leveraging advanced methods to discern usage trends and potential overdose causes [35]. Techniques such as chromatographic analysis and mass spectrometry are pivotal in uncovering these patterns, enabling experts to detect even trace amounts of drugs in biological samples. These methods allow for comprehensive toxicological profiles that illustrate the substance use history of individuals, aiding in the identification of risky behavior or polydrug use patterns. By recognizing these patterns, toxicologists can contribute valuable insights into the circumstances surrounding overdose fatalities [13-27].

The implications of drug interactions on overdose fatalities are profound, as they can significantly alter the toxicological landscape of a case. When drugs interact in unexpected ways, they can increase the risk of fatal outcomes, complicating the determination of the cause of death [7]. Forensic toxicologists must consider these interactions to accurately assess whether a death was accidental, intentional, or due to misuse. This consideration is particularly important in cases where multiple substances are involved, as drug interactions can exacerbate toxicity levels beyond lethal thresholds. By meticulously analyzing these interactions, toxicologists ensure that the conclusions drawn from toxicological reports are as precise and informative as possible, ultimately assisting in the broader efforts of public health and safety [13-27].

Post mortem redistribution

Postmortem redistribution (PMR) is a critical factor influencing the interpretation of toxicological findings, particularly in overdose cases. This phenomenon refers to the postmortem movement of substances from regions of high concentration, such as the liver or gastrointestinal tract, into the bloodstream, leading to artificially elevated drug concentrations in certain samples, such as peripheral blood. PMR complicates the determination of drug levels at the time of death, potentially obscuring the distinction between therapeutic, toxic, and lethal concentrations [23-25].

For example, lipophilic drugs like fentanyl, methadone, and tricyclic antidepressants are particularly prone to PMR due to their chemical properties, which favor redistribution from tissues into the blood. Studies have shown that the extent of redistribution can vary significantly based on factors such as the drug's volume of distribution, the site of blood sampling, and the time elapsed since death [13-27]. Peripheral blood, often preferred for postmortem analysis, is less affected by PMR compared to central sites such as the heart, but discrepancies can still arise if sampling protocols are not rigorously standardized.

In overdose investigations, understanding PMR is essential to avoid misinterpretation of results. For instance, artificially elevated drug levels in postmortem blood may lead to overestimation of the substance's contribution to the cause of death. Conversely, drugs that undergo significant redistribution might be underestimated in their role if only central blood concentrations are considered. To mitigate these challenges, complementary analysis of multiple biological matrices, such as liver tissue, vitreous humor, or even brain tissue, is often employed [23-26]. These matrices can provide additional insights into the distribution and metabolism of substances, helping to clarify the timeline and extent of drug exposure.

By integrating a detailed understanding of PMR into toxicological evaluations, forensic experts can provide more accurate interpretations of postmortem drug concentrations. This is particularly important in complex overdose cases involving polydrug use, where interactions between substances and the dynamics of redistribution may further complicate the analysis. Addressing PMR systematically ensures that toxicological findings more accurately reflect the circumstances leading to death, ultimately enhancing the reliability of forensic investigations.

Stability of substances

The stability of substances in biological matrices is a fundamental factor influencing the reliability of toxicological results. Various drugs and their metabolites can undergo chemical and enzymatic changes after collection, particularly in postmortem samples, where decomposition processes and environmental conditions exacerbate instability. Factors such as temperature, pH, and the presence of enzymatic activity in the matrix can significantly affect the concentration of substances, leading to potential degradation or transformation [13-27].

For instance, benzodiazepines and opiates, commonly encountered in overdose cases, are generally stable in biological matrices under proper storage conditions, such as refrigeration or freezing [13-27]. However, certain substances, such as cocaine, are known to degrade rapidly due to hydrolysis into benzoylecgonine, making timely collection and preservation of samples critical. Similarly, synthetic opioids like fentanyl exhibit relatively high stability, but their metabolites may degrade faster, influencing the interpretation of toxicological results.

The choice of biological matrix also plays a pivotal role in substance stability. Blood, often the primary sample in toxicology investigations, is prone to changes due to the presence of enzymes and ongoing postmortem redistribution. Urine, in contrast, provides a more stable environment for detecting metabolites over extended periods but offers limited information on the concentration of substances at the time of death [14-26]. Hair samples, while less commonly used in acute cases, are highly stable over time and can provide insights into long-term substance use. Tissues such as the liver or brain may also be analyzed to evaluate stability, especially for lipophilic drugs that accumulate in these matrices.

To ensure the reliability of toxicological results, strict protocols must be followed for the collection, storage, and transport of samples. Immediate refrigeration or freezing is often necessary to minimize degradation. Additionally, analyzing multiple matrices and comparing findings can help corroborate results and account for any potential instabilities in a single matrix. Advanced analytical techniques, such as LC-MS/MS, further enhance the detection of substances and their metabolites, even when partial degradation has occurred [28-32].

Addressing the stability of substances in biological matrices is crucial for the accurate interpretation of toxicological findings. By mitigating factors that compromise stability, forensic experts can provide more reliable insights into the presence and role of substances in overdose deaths, strengthening both medical and legal conclusions.

Alternative matrices

The growing importance of alternative biological matrices, such as hair, oral fluid, and nails, in forensic toxicology has expanded the field's ability to detect and interpret substance use, particularly in long-term cases [28, 29]. These matrices complement traditional samples like blood and urine, offering distinct advantages in specific forensic scenarios. Hair analysis has become a key tool for detecting long-term substance use [31]. Due to its unique growth pattern and incorporation of drugs into the keratin structure, hair provides a chronological record of exposure, allowing for the retrospective detection of substances over weeks, months, or even years, depending on hair length. For example, hair testing has proven particularly useful in documenting chronic opioid or amphetamine use, as well as identifying patterns of abuse in cases of suspected workplace substance misuse or child custody disputes. Its stability over time also makes it invaluable in postmortem cases where other matrices may have degraded.

Oral fluid has gained prominence for its non-invasive collection and ability to detect recent drug use. The matrix reflects the free, pharmacologically active fraction of substances present in the bloodstream, making it ideal for determining impairment in cases of suspected drug-facilitated crimes or driving under the influence (DUI) [28, 29]. Despite its limited detection window compared to hair, oral fluid is well-suited for identifying substances like cannabis, cocaine, or synthetic cannabinoids within hours to a few days after use. Advances in collection devices and analytical methods, such as LC-MS/MS, have further enhanced the reliability of oral fluid testing.

Nail analysis represents another alternative matrix with significant forensic potential. Similar to hair, nails incorporate drugs into their keratinized structure and provide a long-term record of exposure, although the timeline is less precise due to slower growth rates. Nails have been used to detect substances such as methamphetamine, cocaine, and benzodiazepines, often in cases where other matrices are unavailable or compromised. Additionally, nail testing can be particularly effective for monitoring chronic exposure to toxic substances like arsenic or other environmental toxins.

The integration of alternative matrices into forensic toxicology enhances the ability to detect substance use across various timelines and contexts. As analytical technologies continue to advance, the adoption of these matrices will further improve the scope and accuracy of toxicological investigations, particularly in complex cases involving long-term substance use or limited sample availability (Table 2).

**Table 2:** Comparative analysis of biological matrices Table credits: authors Matteo A. Sacco and Isabella Aquila PMR - postmortem redistribution; NPS - novel psychoactive substances; DUI - driving under the influence

Biological matrix	Advantages	Disadvantages	Detection window	Applications
Blood	Reflects concentration at death	Prone to PMR	Hours to days	Acute toxicity, overdose cases
Urine	Long detection window	Limited in concentration analysis	Days to weeks	Routine drug screening
Hair	Long-term detection	Susceptible to external contamination	Months to years	Chronic substance use, NPS
Oral fluid	Non-invasive, reflects recent use	Short detection window	Hours to days	DUI cases, drug-facilitated crimes
Nails	Long-term exposure	Slower incorporation	Weeks to months	Chronic exposure, environmental toxins

While these alternative matrices offer distinct advantages, their interpretation requires careful consideration of pharmacokinetics, incorporation mechanisms, and individual variability. For example, external contamination of hair and nails must be excluded through rigorous washing protocols and analytical controls. In oral fluid, drug concentration levels can be influenced by pH, salivary flow rate, and collection method, necessitating standardized procedures to ensure reliable results.

Cerebrospinal fluid (CSF) can be valuable for detecting drugs that have crossed the blood-brain barrier, making it particularly useful in cases of central nervous system toxicity or postmortem investigations. Skeletal tissue, especially bone and bone marrow, serves as a long-term reservoir for drug and toxin accumulation, proving essential in cases of advanced decomposition or when traditional biological matrices are unavailable. Sweat analysis, though less commonly used, provides a non-invasive means of monitoring drug use over an extended period, making it useful for workplace drug testing, rehabilitation programs, and continuous exposure assessments.

The inclusion of these alternative matrices enhances the scope of forensic toxicology, allowing for more comprehensive toxicological investigations, especially in cases where conventional samples are unavailable or degraded.

Legal and forensic implications

The role of toxicology in legal investigations of overdose deaths is indispensable [4]. Forensic toxicologists meticulously analyze biological specimens to uncover the presence and concentration of drugs or toxins in the deceased individual, thereby providing critical data that can influence legal outcomes [28]. These analyses help establish whether the drugs were present in lethal amounts or if there were interactions between multiple substances that could have contributed to the fatality. By collaborating closely with law enforcement and medical examiners, toxicologists ensure that the investigations are thorough and scientifically sound, ultimately aiding in the accurate classification of deaths as accidental, suicidal, or homicidal. This collaboration is crucial for building a comprehensive case that can withstand scrutiny in a court of law.

Toxicology findings profoundly influence court proceedings related to overdose deaths. The data provided by toxicological analyses can serve as concrete evidence in legal cases, helping to substantiate claims of negligence, malpractice, or criminal activity [28]. For instance, the detection of illicit drugs in an individual's system can support allegations of drug distribution or abuse. Conversely, the absence of expected therapeutic drugs might suggest non-compliance with medical prescriptions or wrongful distribution by healthcare providers. Toxicologists often present their findings in court, where their expert testimony can elucidate complex scientific concepts to judges and juries, ensuring that the evidence is correctly interpreted and applied in the judicial process. This expert involvement underscores the importance of forensic toxicology as a bridge between science and law, facilitating informed decisions that can lead to justice being served.

Maintaining the chain of custody for evidence is of paramount importance in toxicology investigations of overdose deaths. This process involves the meticulous documentation of the collection, transfer, and analysis of biological specimens to ensure their integrity and authenticity [38]. A well-documented chain of custody provides assurance that the evidence has not been tampered with or contaminated, which is essential for its admissibility in court. It is crucial that each individual who handles the specimens records their actions accurately, as any lapse can lead to challenges in the legal proceedings. This rigorous documentation process not only protects the evidence but also upholds the credibility of the forensic toxicology findings. By adhering to strict protocols, toxicologists and investigators can provide reliable data that withstand legal scrutiny, thereby reinforcing the validity of the investigation and the subsequent legal outcomes.

Toxicology in public health and policy

Toxicology data plays a crucial role in shaping public health strategies by providing detailed insights into the substances involved in overdose deaths. These investigations allow health authorities to understand the prevalence and patterns of drug use within communities, facilitating the development of targeted interventions. For instance, by analyzing toxicology reports, public health officials can identify emerging drug threats and tailor their response strategies accordingly [39]. Furthermore, toxicology data contribute to the design of surveillance systems that track overdose trends and help in the allocation of resources to areas most affected by drug-related issues. This data-driven approach ensures that public health measures are informed, timely, and effective.

The impact of toxicology investigations extends beyond public health strategies and significantly influences policy-making for drug regulation and prevention. By providing empirical evidence on the substances contributing to overdose fatalities, toxicology data informs legislative bodies on the need for regulatory changes and new policies. For example, the identification of dangerous synthetic opioids through toxicological analysis can lead to stricter regulations and the scheduling of these substances [40]. Additionally, policymakers can use toxicology data to support the implementation of harm reduction programs, such as supervised consumption sites or the distribution of naloxone, to prevent overdose deaths. Thus, toxicology investigations serve as a foundational element for evidence-based policy-making in the realm of drug regulation and prevention.

Toxicology investigations also play a vital role in enhancing community awareness and education initiatives about the risks of drug use. By disseminating information derived from toxicology reports, communities can be better informed about the specific substances and combinations that pose the greatest threat. This knowledge empowers local organizations and educational institutions to develop targeted communication strategies that address the dangers of polydrug use and the potential for accidental overdoses [41]. Moreover, toxicology data can be used to design educational programs that teach individuals how to recognize the signs of overdose and take appropriate action, ultimately reducing the stigma associated with seeking help. Incorporating toxicology insights into community education efforts ensures that these programs are grounded in scientific evidence and tailored to address local drug-related challenges.

Challenges and future directions in toxicology

Current toxicology practices face several limitations that can hinder effective investigations of overdose deaths. One significant challenge is the lack of standardized methodologies across different laboratories, which can lead to inconsistencies in results and interpretations [11]. This variability can complicate collaborative efforts among medical examiners, toxicologists, and law enforcement agencies, ultimately affecting the accuracy of drug-related death investigations [4]. Additionally, many toxicology labs still rely on outdated technologies that struggle to keep pace with the rapid emergence of new synthetic drugs and their analogs. As a result, detecting and accurately identifying these substances in biological samples remains a formidable task. Furthermore, postmortem redistribution of drugs within the body can obscure toxicological findings, complicating the determination of cause and manner of death [10]. Together, these limitations highlight the urgent need for advancements in toxicological methods and practices.

Emerging technologies and methods are poised to revolutionize toxicological analysis, offering new opportunities for improving the accuracy and efficiency of overdose investigations. The adoption of advanced analytical techniques, such as liquid chromatography-tandem mass spectrometry (LC-MS/MS), allows for the precise detection of a broader range of substances, including novel psychoactive compounds [40]. These technologies not only enhance sensitivity and specificity but also reduce the time required for analysis, facilitating more timely and reliable results. Additionally, the development of bioanalytical tools, such as 3D liver models, offers promising avenues for better understanding drug metabolism and toxicity [31]. These models can bridge the gap between in vitro and in vivo studies, providing more accurate predictions of how drugs behave in the human body. As these technologies continue to evolve, they hold the potential to significantly enhance the capabilities of toxicology laboratories.

**Figure 2:**
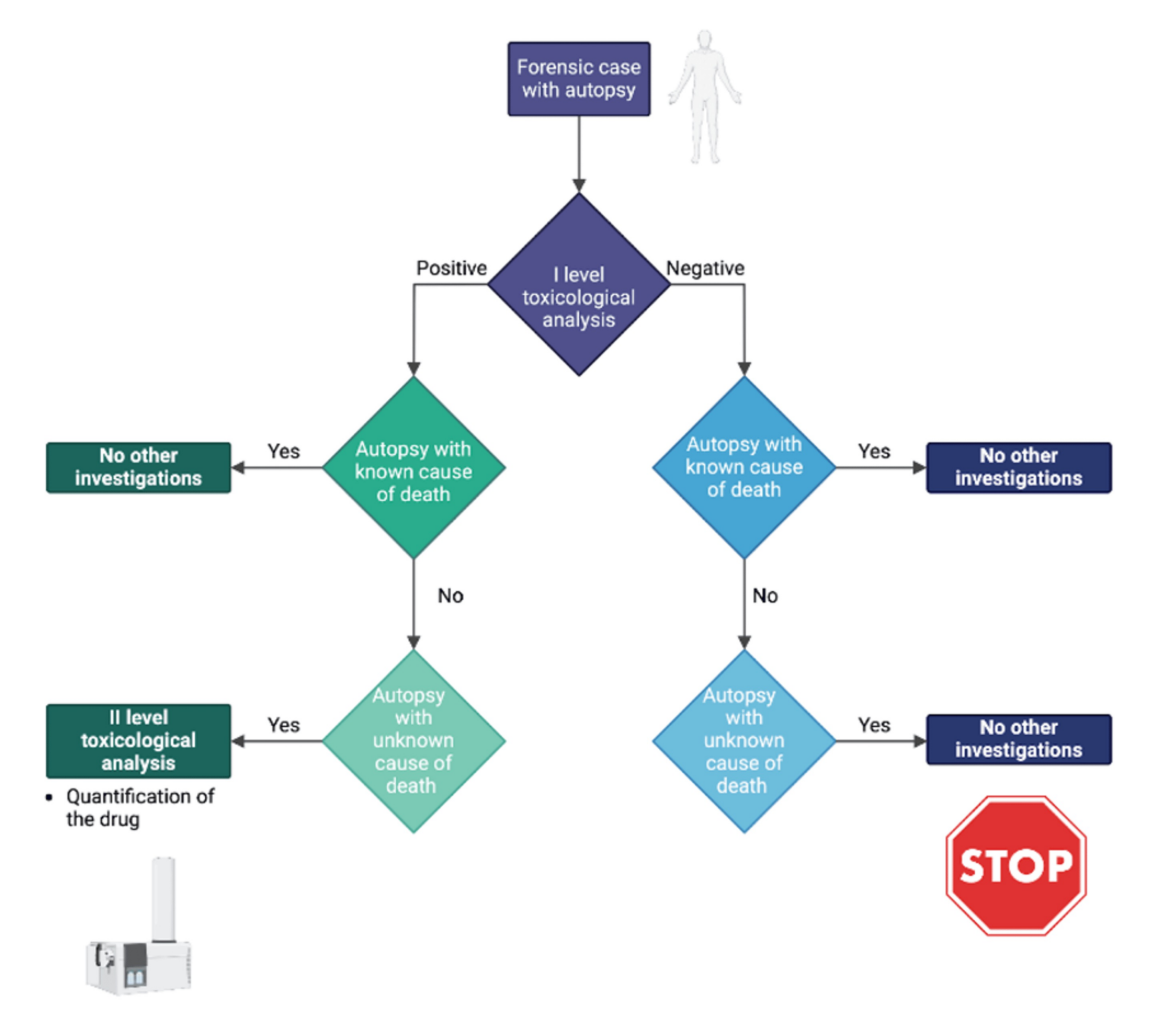
Algorithm for carrying out toxicological investigations Created with biorender.com

Looking to the future, several trends and potential advancements in toxicology are expected to address the current challenges and expand the field's capabilities. One promising trend is the integration of machine learning and artificial intelligence into toxicological data analysis. These technologies can process large datasets more efficiently, identifying patterns and correlations that may be missed by traditional methods [40]. Moreover, the ongoing miniaturization and automation of analytical instruments promise to increase the accessibility and affordability of comprehensive toxicological testing [36]. This democratization of technology could enable smaller laboratories to adopt cutting-edge techniques, improving the overall standard of toxicological investigations. Furthermore, the continued exploration of alternative matrices, such as hair and oral fluid, could provide additional insights into drug use history and patterns [41, 42]. As these trends gain momentum, they are likely to drive significant advancements, ultimately enhancing the role of toxicology in public health and safety (Figure 2) [43-48].

## Conclusions

This study highlights the critical role of forensic toxicology in understanding and addressing overdose deaths. By utilizing advanced analytical methods, toxicological investigations provide vital insights into the substances involved, their concentrations, and their impact on the cause of death. The integration of traditional and alternative biological matrices enhances the reliability of findings, while advancements in technology improve detection capabilities. These insights not only inform legal and medical determinations but also contribute to public health strategies aimed at mitigating substance abuse and its consequences. Focusing on accurate interpretation and the implementation of standardized protocols ensures that forensic toxicology remains a cornerstone of modern investigative processes.
